# Is Decoupling GDP Growth from Environmental Impact Possible?

**DOI:** 10.1371/journal.pone.0164733

**Published:** 2016-10-14

**Authors:** James D. Ward, Paul C. Sutton, Adrian D. Werner, Robert Costanza, Steve H. Mohr, Craig T. Simmons

**Affiliations:** 1 School of Natural and Built Environments, University of South Australia, GPO Box 2471, Adelaide, SA, 5001, Australia; 2 Department of Geography, University of Denver, Denver, Colorado, 80208, United States of America; 3 School of the Environment and National Centre for Groundwater Research and Training, Flinders University, GPO Box 2100, Adelaide, SA, 5001, Australia; 4 Crawford School of Public Policy, The Australian National University, Acton, ACT, 2601, Australia; 5 Institute for Sustainable Futures, University of Technology Sydney, PO Box 123, Broadway, NSW, 2007, Australia; Universidad de la Republica Uruguay, URUGUAY

## Abstract

The argument that human society can decouple economic growth—defined as growth in Gross Domestic Product (GDP)—from growth in environmental impacts is appealing. If such decoupling is possible, it means that GDP growth is a sustainable societal goal. Here we show that the decoupling concept can be interpreted using an easily understood model of economic growth and environmental impact. The simple model is compared to historical data and modelled projections to demonstrate that growth in GDP ultimately cannot be decoupled from growth in material and energy use. It is therefore misleading to develop growth-oriented policy around the expectation that decoupling is possible. We also note that GDP is increasingly seen as a poor proxy for societal wellbeing. GDP growth is therefore a questionable societal goal. Society can sustainably improve wellbeing, including the wellbeing of its natural assets, but only by discarding GDP growth as the goal in favor of more comprehensive measures of societal wellbeing.

## Introduction

The perpetually growing economy is generally regarded as a viable and desirable societal objective [1–4]. Whilst ‘infinite growth’ may not be the words used to characterize and exhort a perpetually growing economy, they are nonetheless an accurate characterization of the objective. The words in current fashion for defending the viability of a perpetually growing economy are phrases such as ‘green growth’, ‘dematerialization’, and ‘decoupling’ [5–9]. The decades old question ‘*Is economic growth environmentally sustainable*?’ remains contested despite its apparent simplicity. The Limits to Growth [10] was a seminal work that warned of the consequences of exponential growth with finite resources. The World3 models underpinning the Limits to Growth analysis were validated using actual data after twenty and thirty years [11,12]. A further independent evaluation of the projections of the World3 models showed that our actual trajectory since 1972 has closely matched the ‘Business as Usual’ scenario [13]. Increasing recognition of the causes and consequences of climate change have generated a great deal of doubt regarding the feasibility of simultaneously pursing economic growth and preventing and/or mitigating climate change [14–18]. Contemporary work in this broad area of assessing anthropogenic impact on the planet suggests that several ‘Planetary Boundaries’ have been crossed [19].

The question as to whether human society can decouple economic growth–defined as growth in Gross Domestic Product (GDP)–from environmental impacts has not been settled. The decoupling debate itself is polarized with a preponderance of neo-classical economists on one side (decoupling is viable) and ecological economists on the other (decoupling is not viable) [20]. The divide over the compatibility of economic growth and environmental limits extends into the general public [2] with substantial polarization in ideas of decoupling, dematerialization, and limits to growth.

Settling the debate has far reaching policy implications. Decoupling is increasingly being described in popular press as a viable policy objective [21,22]. Decoupling has been incorporated into international indicators of sustainable development [23] and policy objectives such as the United Nations’ ‘Sustainable Development Goals’ [24]. If decoupling is possible, then these policies are valid sustainable goals; however, if decoupling is shown to be nonviable then society will need to shift away from the current ‘infinite growth’ model.

**Fig 1:**
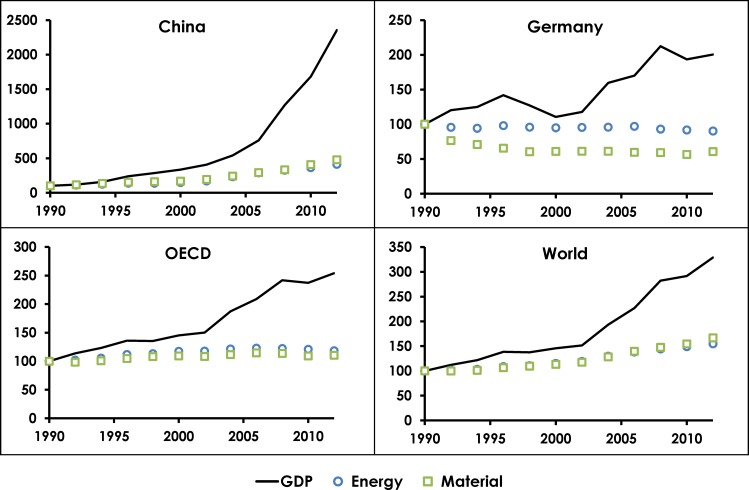
Recent trends in real GDP, total energy use and total material use for China, Germany, OECD and the World. Data are normalized to 100 in the year 1990.

Decoupling is defined as either ‘relative’ (aka ‘weak’) or ‘absolute’ (aka ‘strong’). Relative decoupling refers to higher rates of economic growth than rates of growth in material and energy consumption and environmental impact. As a result, relative decoupling implies a gain in efficiency rather than removal of the link between impact and GDP. Recent trends (1990 to 2012) for GDP [25], material use [26] and energy use [27] in different countries and regions exhibit different behavior (Fig 1). In China, relative decoupling has occurred as GDP (market prices, in current US$) increased by a factor of more than 20 over the 22-year period, while energy use rose by a factor of slightly more than four and material use by almost five. Germany, meanwhile, exhibited slower GDP growth than China, but at the same time reduced energy use by 10% and total material use by 40%. The OECD follows a similar story to Germany, albeit with flat rather than falling energy and material consumption. Although Germany and the OECD give hope that absolute decoupling may be achievable, at the global level we see only relative decoupling with energy and material use increasing by 54% and 66% over the 22 years, respectively.

Similar evidence to that in Fig 1, showing apparent decoupling of GDP from specific resources, has been shown throughout much of the OECD [28]. However, there are several limitations to the inference of decoupling from national or regional data. There are three distinct mechanisms by which the illusion of decoupling may be presented as a reality when in fact it is not actually taking place at all: 1) substitution of one resource for another; 2) the financialization of one or more components of GDP that involves increasing monetary flows without a concomitant rise in material and/or energy throughput, and 3) the exporting of environmental impact to another nation or region of the world (i.e. the separation of production and consumption). These illusory forms of decoupling are described with respect to energy by our colleague [29].

An additional mechanism of decoupling is associated with growing inequality of income and wealth, which can allow GDP to grow overall while the majority of workers do not see a real gain in income [30]. This growth in inequality can manifest as higher GDP without a proportional increase in material and energy flow (i.e. relative decoupling) when a wealthy minority of the population derives the largest fraction of GDP growth but does not necessarily increase their level of consumption with as much demand for energy and materials [31]. In such cases, at the aggregate level decoupling would be observed, but it is doubtful that such unequal sharing of growth in GDP represents an improvement in wellbeing.

At the World aggregate level, Fig 1 shows relative decoupling with a growing gap between GDP and resource consumption. In the context of reaching planetary boundaries and global environmental limits, however, relative decoupling will be insufficient to maintain a GDP growth-oriented human civilization. The only way to achieve truly sustainable growth would be via permanent absolute decoupling. Absolute decoupling theoretically occurs when environmental impacts are reduced while economic growth continues. While relative decoupling has been observed in multiple countries, absolute decoupling remains elusive [32–34]. According to one study [35] no country has achieved absolute decoupling during the past 50 years. Another study [36] reports that population growth and increases in affluence are overwhelming efficiency improvements at the global scale. They find no evidence for absolute reductions in environmental impacts, and little evidence to date even for significant relative decoupling.

It should be noted that technological advances can lead to absolute decoupling for specific types of impact [37]. It is possible, for instance, to substitute a polluting activity with a non-polluting activity, and notable examples have included the removal of tetraethyl lead from automotive fuel and CFCs from refrigerants and propellants. It is also possible to envisage a scenario in which GDP growth is decoupled from the use of fossil fuels and related CO_2_ emissions by switching to 100% renewable energy, but this is not the same as decoupling GDP growth from energy use. In the context of this study, we are primarily interested in fundamental resources (matter and energy) as the foundations of economic activity.

In the current paper, we show that decoupling scenarios can be interpreted using an easily understood model of economic growth and environmental impact. The simple model was calibrated against published data derived from sophisticated predictive studies of decoupling, and used to develop a long-term prognosis of environmental impact under continued GDP growth. The results are then used to draw conclusions about the long-term viability of GDP growth as a societal goal.

## Model Derivation

We use a simple mathematical model to develop insights into decoupling behavior. We start with the IPAT equation [38–40], which is a basic formulation of environmental impact *I* as a function of economic activity:

I=PAT (1)

where *P* is population, *A* is affluence (GDP per capita in $/person/year) and *T*, as originally formulated, represents “technology”. More precisely, however, *T* should be viewed as the economic intensity of a particular resource or pollutant, and therefore both *T* and *I* will have different units depending on which resource or pollutant is considered. For energy, appropriate units for *T* may be joules per $ of GDP; for material use *T* may be kilograms per $ of GDP. The terms *T* and *I* can–and should–thus be evaluated separately, with appropriate units, for individual resources such as farming land, fresh water and energy resources, and/or pollutant emissions such as sulphur dioxide, lead, or carbon dioxide.

To test the hypothesis that continual GDP growth can be sustained, we only require a scenario in which GDP increases exponentially. The economy (as GDP) can thus be simplified to *G* = *PA*, leading to *I*_*j*_ = *GT*_*j*_ where *I*_*j*_ and *T*_*j*_ are the impact and economic intensity, respectively, of resource or pollutant *j*. A simple case is one in which both population (*P*) and affluence (*A*) are increasing exponentially, but other combinations (e.g. stationary population with rising affluence) could achieve the same result of rising GDP. There are, of course, scenarios that could lead to falling GDP (e.g. declining population with constant affluence, or both falling) but our investigation is directed explicitly at testing the sustainability of continual economic growth as a societal goal. As such, we assume *G* at time *t* is given by:

$$G\left(t\right)=G_{0}e^{kt}$$ (2)

where *G*_0_ is the initial GDP at time *t* = 0, and *k* is the growth rate per year. Hence, impact (for resource or pollutant *j*) over time is given as:

$$I_{j}\left(t\right)=G_{0}e^{kt}T_{j}$$ (3)

If there is no technological change to reduce a particular impact (i.e. *T*_*j*_ = constant; no decoupling), the use of resources or pollutant emissions will rise exponentially, in keeping with GDP growth. For absolute decoupling from resource or pollutant *j*, *T*_*j*_ must decrease exponentially at the same rate as GDP growth such that *I*_*j*_ remains constant in time, i.e.:

$$T_{j}=T_{j,0}e^{-kt}$$ (4)

where *T*_*j*,0_ is the initial level of economic intensity of resource or pollutant *j*.

Put simply, absolute decoupling from resource or pollutant *j* requires *T*_*j*_ to decrease by at least the same annual percentage as the economy is growing. For example, if *k* = 0.03 (steady 3% p.a. economic growth), *T*_*j*_ must reduce 20-fold over 100 years, 100-fold over 150 years, and 500-fold over 200 years, and continue this trend of exponential reduction as long as the economy is growing. If *T*_*j*_ were to decrease at a faster rate than GDP growth, the impact *I*_*j*_ would decline.

For non-substitutable resources such as land, water, raw materials and energy, we argue that whilst efficiency gains may be possible, there are minimum requirements for these resources that are ultimately governed by physical realities: for instance the photosynthetic limit to plant productivity and maximum trophic conversion efficiencies for animal production govern the minimum land required for agricultural output; physiological limits to crop water use efficiency govern minimum agricultural water use, and the upper limits to energy and material efficiencies govern minimum resource throughput required for economic production. Therefore a more appropriate formulation of Eq (4) is to allow *T*_*j*_ to decrease to an ultimate value, *T*_*ult*_ ≥ 0, as follows:

$$T_{j}=T_{j,ult}+\left(T_{j,0}-T_{j,ult}\right)e^{-r_{j}t}$$ (5)

where *T*_*j*,*ult*_ is the ultimate resource use intensity, and *r*_*j*_ is the rate of exponential decline, for resource or pollutant *j*. In cases where decoupling is occurring, *T*_*j*,*ult*_ < *T*_*j*,0_. However, cases where resource use intensity is increasing towards an upper limit can be accommodated with *T*_*j*,*ult*_ > *T*_*j*,0_.

The nature of decoupling behavior for different types of resource can be readily predicted from the relationships between *r*_*j*_, *k*, *T*_*j*,*ult*_ and *T*_*j*,0_ as summarized in Table 1. It is only those resources or pollutants for which *r*_*j*_ > *k* and *T*_*j*,*ult*_ < *T*_*j*,0_ (i.e. efficiency gains are possible and can be achieved faster than the economy is growing) that a period of decoupling can be expected.

**Table 1:** Summary of resource conditions and resultant decoupling behavior.

Conditions	Behaviour
*r*_*j*_ > *k*, *T*_*j*,*ult*_ = 0	Absolute decoupling (*I*_*j*_ declines and does not grow again)
*r*_*j*_ *= k*, *T*_*j*,*ult*_ = 0	Absolute decoupling (*I*_*j*_ stable and does not grow or decline)
0 < *r*_*j*_ *< k*, *T*_*j*,*ult*_ = 0	Relative decoupling (*I*_*j*_ grows slower than GDP)
*r*_*j*_ > *k*, 0 < *T*_*j*,*ult*_ < *T*_*j*,0_	Temporary decoupling (*I*_*j*_ declines for a period, then resumes growth once *T*_*j*_ stabilizes, and tends towards a growth rate equal to GDP growth)
0 < *r*_*j*_ *≤ k*, 0 < *T*_*j*,*ult*_ < *T*_*j*,0_	Temporary decoupling (*I*_*j*_ grows slower than GDP for a period, then tends towards a growth rate equal to GDP growth)
*r*_*j*_ > 0, *T*_*j*,*ult*_ > *T*_*j*,0_	No decoupling; resource use intensity increasing.

## Model Application

A recent predictive study [41] concluded that Australia could–through adoption of specific policies–*“achieve strong economic growth to 2050 … in scenarios where environmental pressures fall or are stable”* (this study is referred to as “H-D” hereafter). That paper summarized the results of a significant project, the *2015 Australian National Outlook* [42] published by the Commonwealth Science and Industrial Research Organisation (CSIRO), and represents a high-profile, contemporary study in decoupling. In all of their modelled scenarios, both population and gross national income per capita increased. In their strong abatement scenario (called “Stretch”), various forms of decoupling behavior were predicted. We will use the Stretch scenario from H-D as a case study in decoupling, and will use Eq (5) to further explore the behavior of energy and material use and implications for longer-term impact. The data used in H-D’s historical and projected scenarios are all available in their Supplementary Information files that accompanied their original publication. Likewise, the data and model results for the following analysis can all be found in the S1 File accompanying this paper.

We begin by calibrating the decline rate *r*_*j*_ from Eq (5) against H-D’s historical energy use (*j* = 1) and material extraction (*j* = 2). The term *T*_*j*_ for each resource is found by dividing GDP (*G*) by resource use (*I*_*j*_) on a yearly timestep. For energy, units are MJ per thousand $AUD (2010). *T*_1,0_ = 3.783, equal to the value of *T*_1_ in the year 1980. The ultimate resource use intensity *T*_1,*ult*_ is unknown, and depends on future technological advances. To account for this uncertainty, three scenarios are adopted: high decoupling (*T*_1,*ult*_ = 0.25 *T*_1_(2010) = 0.704), medium decoupling (*T*_*ult*_ = 0.5*T*_1_(2010) = 1.408) and low decoupling (*T*_*ult*_ = 0.75 *T*_1_(2010) = 2.113). Under each of the three *T*_1,*ult*_ scenarios, Eq (5) is used to predict *T*_1_, and is calibrated by varying the single unknown parameter (the decline rate, *r*_1_) to give the best fit between our *T*_1_ (predicted) and *T*_1_ (historical) derived from H-D’s data. The calibration was performed using the free statistical package “R” (https://www.r-project.org) with the in-built Non-Linear Least Squares function.

**Fig 2:**
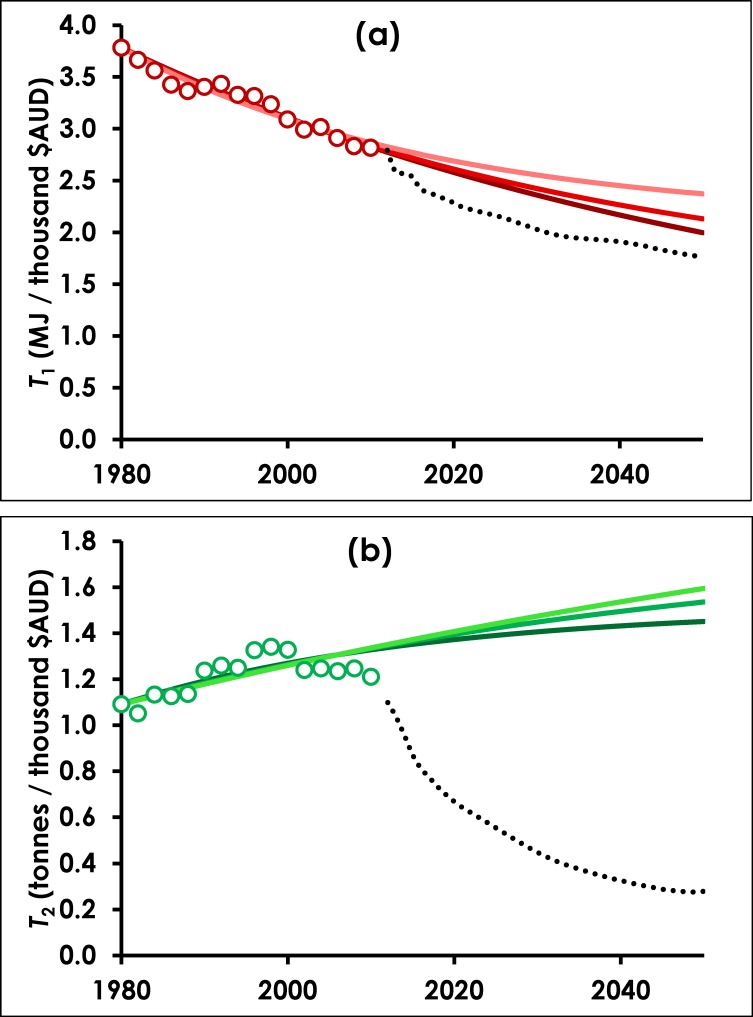
**Calibrated model against (a) final energy demand and (b) material extractions from 1980–2010.** Historical data (circles) are from H-D. Dark, medium and light solid lines represent low, middle and high values of *T*_*j*,*ult*_. Dotted black lines are the projections of *T*_*j*_ inferred from H-D’s Stretch scenario.

All three scenarios reproduce the observed downward trend in *T*_1_. Calibrated declines rates with standard error (in brackets) were 1.24% (± 0.05%), 1.70% (± 0.07%) and 2.66% (± 0.13%) for high, medium and low respectively. The result is a simple calibrated model that can be used to project forward based on recent trends, for the purpose of comparing against the more complex modelling scenarios from H-D. The results are shown in Fig 2(A), with the modelled *T*_1_ values projected to 2050. The decoupling predicted by H-D is also shown (taking *T*_1_ as their predicted impact divided by their predicted GDP). Clearly H-D predict stronger decoupling than our strongest case. In terms of the simplified IPAT model, even for our most optimistic *T*_1,*ult*_ scenario, this implies a change to a greater decline rate than can be obtained merely by calibrating against historical trends.

In the case of material extraction, *T*_2_ has been increasing over recent years; in other words, according to H-D’s historical data, material use has not been on a decoupling trajectory in Australia. This is not unexpected, reflecting Australia’s strong dependence on its extractive industries. However, it means that in order to obtain a good fit to historical data, *T*_2,*ult*_ must be greater than *T*_2,0_. The units of *T*_2_ are tonnes of material extracted per thousand $AUD (2010), and *T*_2,0_ = 1.091 in the year 1980. Three scenarios are adopted as an upper bound to future material intensity: low (*T*_2,*ult*_ = 1.25*T*_2_(2010) = 1.513), medium (*T*_2,*ult*_ = 1.50*T*_2_(2010) = 1.815) and high (*T*_2,*ult*_ = 2.00*T*_2_(2010) = 2.420). As before, the model is calibrated by finding the decline rate *r*_2_ for each *T*_2,*ult*_ scenario. The scenarios calibrate well in all three scenarios with *r*_2_ = 2.75% (± 0.47%), 1.36% (± 0.22%) and 0.68% (± 0.11%) for low, medium and high *T*_2,*ult*_ scenarios respectively. Fig 2(B) shows the historical and modelled *T*_2_ values, plus H-D’s projection to 2050. The profound deviation from long-term trends reflects the assumptions embedded within H-D’s Stretch scenario, which anticipates major policy changes and a shift toward very different forms of production.

**Fig 3:**
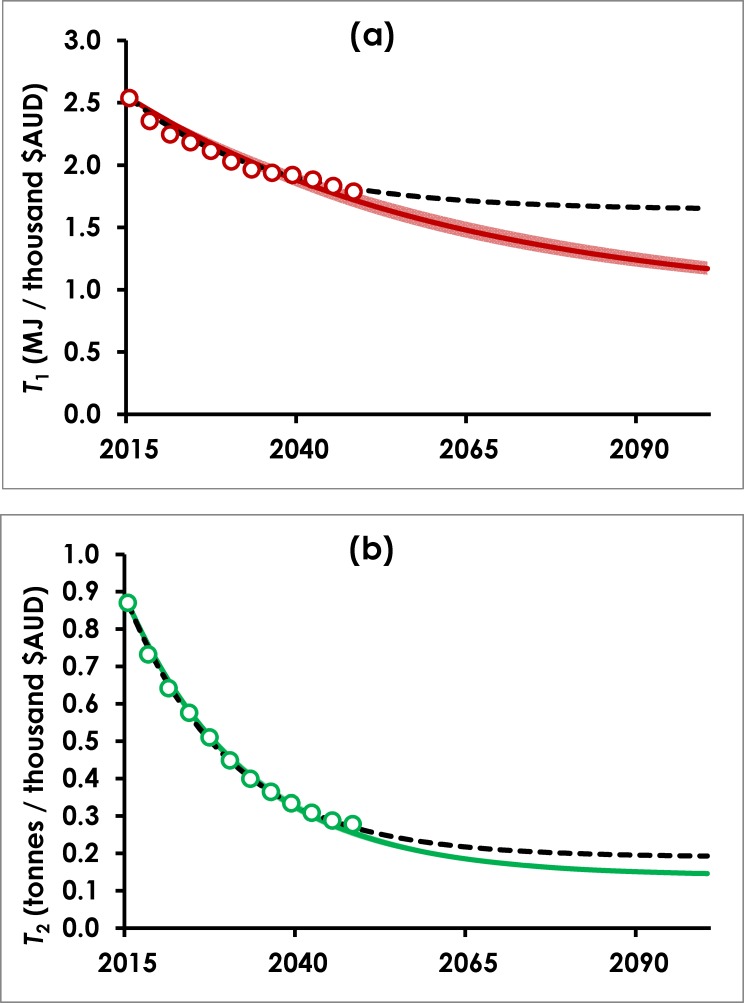
**Calibration of resource use intensity and projection to 2100, for (a) final energy demand, and (b) material extractions.** Circles represent H-D values for *T*_*j*_ (taken as *I*_*j*_ / GDP) plotted every three years, and solid coloured lines represent *T*_*j*_ modelled by Eq (5), calibrating *r*_*j*_ and setting *T*_*j*,*ult*_ = 0.5*T*_*j*_(2050). Around each solid line is a coloured band (most visible for (a) energy) representing the 95% confidence interval for *r*_*j*_. Dashed black lines represent modelled *T*_*j*_ when both *r*_*j*_ and *T*_*j*,*ult*_ are calibrated.

The results of calibrating Eq (5) to historical data are inconclusive; uncertainty in *T*_1,*ult*_ and *T*_2,*ult*_ makes long-term projections unreliable. In any case, historical observations of decoupling at national levels are fraught, for the reasons articulated earlier. We conclude that simplistically extrapolating historical trends is not a reliable technique for projecting future decoupling behavior. Moreover, the sophisticated analysis of H-D suggests that deviations from historical trends in *T*_*j*_ may be plausible, as shown in Figs 2 and 3. Hence from here onward we will focus on the Stretch scenario from H-D and assume it is a plausible future of rapid technological development and proactive policy settings, which could lead to rapid decoupling from energy and material use.

In order to re-calibrate Eq (5) to create a more useful long-term projection, we use the period 2015–2050 in H-D’s Stretch scenario. The scenario already contains embedded assumptions regarding strong efficiency gains (30% drop in energy intensity and almost 70% drop in material intensity by 2050). As with the historical calibration, *T*_1_ and *T*_2_ are taken as H-D’s predicted energy and material use, *I*_1_ and *I*_2_ respectively, divided by their predicted GDP. We adopt a single *T*_*j*,*ult*_ scenario for each resource, and arbitrarily assume resource use intensity can be reduced to 50% of the *T*_*j*_ attained by H-D’s model in 2050, giving values of *T*_1,*ult*_ = 0.881 MJ per thousand $AUD and *T*_2,*ult*_ = 0.139 tonnes per thousand $AUD. The decline rates *r*_*j*_ are calibrated in the same manner as above.

Fig 3 shows the calibrated model runs, with a projection to 2100. Calibrated decline rates are *r*_1_ = 2.06% (± 0.12%) and *r*_2_ = 5.59% (± 0.11%) for final energy demand and material extraction, respectively. A 95% confidence interval on each *T*_*j*_ prediction is obtained by performing additional model runs using upper and lower values for *r*_*j*_ (mean ± 1.96 × SE); this is included as a coloured band around each solid coloured line in Fig 3, but is only clearly visible on *T*_1_, being too narrow to see on *T*_2_. Finally, to check the appropriateness of our *T*_*j*,*ult*_ scenarios, a further calibration is performed by varying both *r*_*j*_ and *T*_*j*,*ult*_. This allows us to estimate the ultimate resource intensity if technology followed the trend projected by H-D, giving calibrated values *T*_1,*ult*_ = 1.64 (± 0.05) and *T*_2,*ult*_ = 0.19 (± 0.01). From this we can see that our projection is more optimistic than the Stretch scenario of H-D (which was their most optimistic scenario), and we proceed on the basis that our modelled conditions must be considered extremely favorable to decoupling.

**Fig 4:**
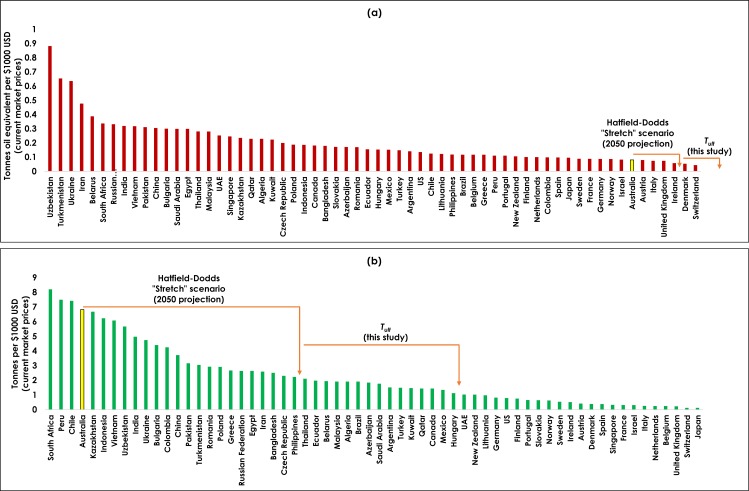
**Reference *T***_***j***_ **values for (a) energy and (b) material use, for Australia and other selected countries.** Derived from GDP [25], total energy use [27] and total material use [26].

Fig 4 shows values of *T*_*j*_ inferred for current energy and total material use across a range of countries, in order to provide some context to the future decoupling scenarios being modelled. It is clear that by this measure, Australia is already one of the most energy-efficient economies in the world. H-D project that by 2050 Australia will improve further, to be on par with Denmark and Sweden today, and our chosen *T*_1,*ult*_ value assumes Australia can ultimately become more energy-efficient (per unit GDP) than any country on the planet today. With respect to material use, H-D project that by 2050 Australia will have completely transformed from being one of the most materially intensive economies today, to being one of mid-range material intensity (by current measures). Our assumption of a 50% further reduction in *T*_2,*ult*_ would place Australia's ultimate material efficiency at a level equivalent to high-income countries today that have relatively low dependence on extractive industries, such as New Zealand. These assumptions can indeed be viewed as an extremely optimistic scenario of future technological improvement.

**Fig 5:**
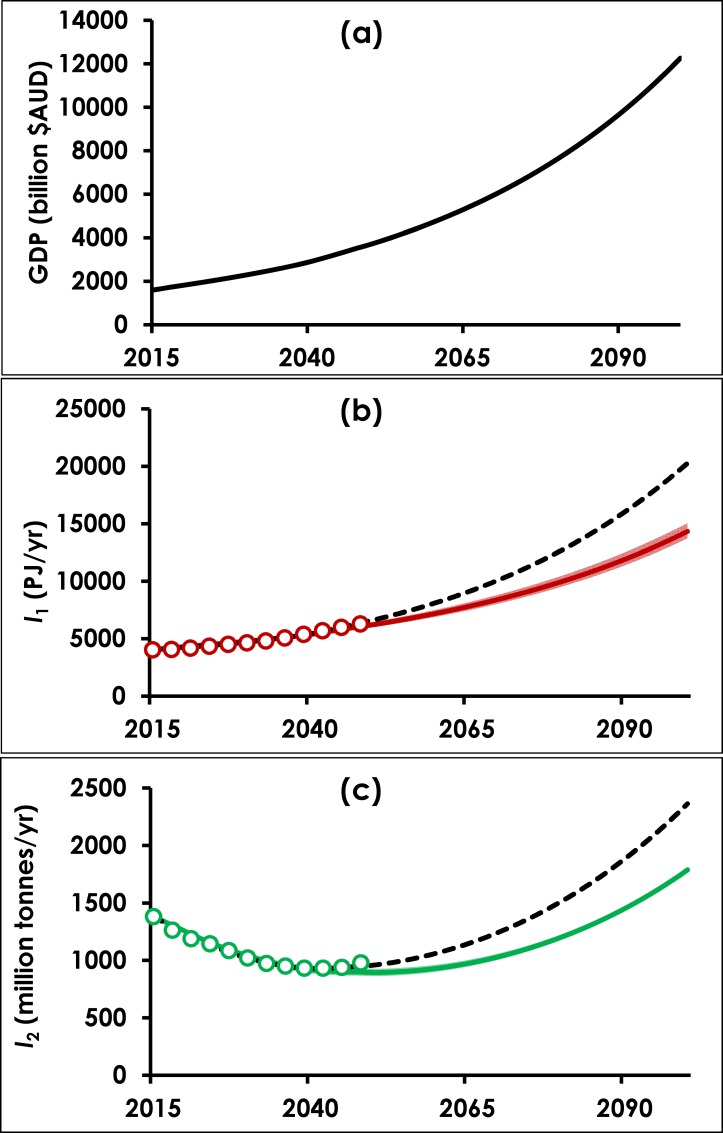
**Projections to 2100 of (a) GDP, (b) final energy demand, and (c) material extractions.** Circles represent H-D values for *I*_*j*_ at 3-year intervals, and solid coloured lines represent *I*_*j*_ modelled using Eqs (3) and (5) on a yearly time step, with *r*_*j*_ calibrated and *T*_*j*,*ult*_ set at 0.5*T*_*j*_(2050). Around each solid line is a coloured band (most visible for (b) energy) representing the 95% confidence interval for *r*_*j*_. Dashed black lines represent modelled *T*_*j*_ when both *r*_*j*_ and *T*_*j*,*ult*_ are calibrated.

The calibrated model can now be used to explore the potential future evolution of resource use under continued economic growth. For this projection, the growth rate *k* = 2.41%, which is the average GDP growth rate from 2015 to 2050 in H-D’s Stretch scenario. Fig 5 shows the modelled projection of impact *I*_*j*_ to 2100. Projected GDP at the end of the century is 7.7 times its 2015 level. Material extractions and final energy demand in 2100 are up 29% (95% confidence interval 28.4–30.7%) and 256% (95% confidence interval 241–273%) respectively, relative to 2015 levels. Considering the embedded optimistic assumptions for *T*_*j*,*ult*_, this result is a robust rebuttal to the claim of absolute decoupling. Fig 5 also includes the projections for *I*_*j*_ using the model in which both *r*_*j*_ and *T*_*ult*_ were calibrated (i.e. most closely reproducing the trends in H-D’s projections). Using that model, energy demand in 2100 would be five times higher than in 2015, and material extraction would rise by 71%.

Importantly, as *T*_*j*_ moves towards a constant value (*T*_*j*,*ult*_), the growth rate of *I*_*j*_ approaches the economic growth rate *k*. Thus, whilst in 2015 the growth rates for material extraction and final energy demand (-1.87% and +1.47% respectively) are all less than the 2.41% economic growth rate, by 2100 these have changed to +2.16% and +1.89% and by 2150, both *I*_1_ and *I*_2_ exhibit growth rates close to the economic growth rate (2.42% and 2.21% respectively).

On the basis of this simple modeling, we conclude that decoupling of GDP growth from resource use, whether relative or absolute, is at best only temporary. Permanent decoupling (absolute or relative) is impossible for essential, non-substitutable resources because the efficiency gains are ultimately governed by physical limits.

## Discussion & Conclusions

Our model demonstrates that growth in GDP ultimately cannot plausibly be decoupled from growth in material and energy use, demonstrating categorically that GDP growth cannot be sustained indefinitely. It is therefore misleading to develop growth-oriented policy around the expectation that decoupling is possible. However, we also note that GDP has been shown to be a poor proxy for societal wellbeing, something it was never designed to measure, and GDP growth is therefore a questionable long-term societal goal in any case. The mounting costs of “uneconomic growth” [43] suggest that the pursuit of decoupling–if it were possible–in order to sustain GDP growth would be a misguided effort.

Society can sustainably improve wellbeing, including the wellbeing of its natural assets, but only by discarding the goal of GDP growth in favor of more comprehensive measures of societal wellbeing [44]. The 17 UN Sustainable Development Goals (SDGs), recently agreed to by all UN countries, represent a much broader conception of the goals of society. These goals include eliminating poverty and hunger, reducing inequality, protecting and restoring the climate, and terrestrial and marine ecosystems. Only one of the 17 goals mentions GDP growth, but it is qualified as “inclusive and sustainable growth”. Certainly, GDP growth over the last several decades has not been inclusive–inequality is getting worse in most countries. For GDP growth to be sustainable it would have to be decoupled from energy and material use and environmental impacts. We have shown that there is little evidence that GDP growth can be decoupled in the long-term (i.e. it is not sustainable).

If GDP growth as a societal goal is unsustainable, then it is ultimately necessary for nations and the world to transition to a steady or declining GDP scenario. We contend that it will be easier to start this transition now while there is still capacity for technological gains, rather than go down the path of decoupling and be forced to make a transition post 2050 when we are closer to the theoretical limits to technological efficiency gains. We argue that now is the time to recognize the biophysical limits, and to begin the overdue task of re-orienting society around a more achievable and satisfying set of goals than simply growing forever [44,45].

## Supporting Information

S1 File Supplementary Data.xlsx. This is a Microsoft Excel spreadsheet containing input data (from H-D) that were used to calibrate the IPAT model for both historical (1980–2010) and projected (2015–2050) data sets. Also shows results of the calibrated model, predicting *T*_*j*_ and *I*_*j*_ thru 2050 (historical calibration) and 2150 (projected calibration).
(XLSX)
